# Astaxanthin Treatment Induces Maturation and Functional Change of Myeloid-Derived Suppressor Cells in Tumor-Bearing Mice

**DOI:** 10.3390/antiox9040350

**Published:** 2020-04-23

**Authors:** Seong Mun Jeong, Yeon-Jeong Kim

**Affiliations:** Laboratory of Microbiology and Immunology, College of Pharmacy, Inje University, Gimhae, Gyeongnam, 50834, Korea; tjdans609@naver.com

**Keywords:** myeloid-derived suppressor cells, astaxanthin, immunosuppression, tumor environment, differentiation

## Abstract

Myeloid-derived suppressor cells (MDSCs) are immature myeloid cells which accumulate in stress conditions such as infection and tumor. Astaxanthin (ATX) is a well-known antioxidant agent and has a little toxicity. It has been reported that ATX treatment induces antitumor effects via regulation of cell signaling pathways, including nuclear factor erythroid-derived 2-related factor 2 (Nrf2) signaling. In the present study, we hypothesized that treatment with ATX might induce maturation of MDSCs and modulate their immunosuppressive activity. Both in vivo and in vitro treatment with ATX resulted in up-regulation of surface markers such as CD80, MHC class II, and CD11c on both polymorphonuclear (PMN)-MDSCs and mononuclear (Mo)-MDSCs. Expression levels of functional mediators involved in immune suppression were significantly reduced, whereas mRNA levels of Nrf2 target genes were increased in ATX-treated MDSCs. In addition, ATX was found to have antioxidant activity reducing reactive oxygen species level in MDSCs. Finally, ATX-treated MDSCs were immunogenic enough to induce cytotoxic T lymphocyte response and contributed to the inhibition of tumor growth. This demonstrates the role of ATX as a regulator of the immunosuppressive tumor environment through induction of differentiation and functional conversion of MDSCs.

## 1. Introduction

Immunotherapy is defined as the treatment of disease by employing the host immune system or immune factors and may complement or substitute for conventional therapies in the treatment of intractable diseases such as cancer [1]. To enable successful cancer immunotherapy, it is important to overcome the immunosuppressive environment [2]. During the induction process of anti-tumor immune responses, tumor antigen (Ag) is taken up by antigen-presenting cells (APCs) and presented to Ag-specific T cells after processing. As a result, Ag-specific T cells are activated to exert their anti-tumor effects [3]. However, the anti-tumor immune response is decreased by immune suppressors present in the tumor environment, such as myeloid-derived suppressor cells (MDSCs), regulatory T cells (Tregs), tumor-associated macrophages, and type II neutrophils [4]. 

MDSCs account for approximately 2%–3% of cells under normal conditions but are multiplied by several orders of magnitude and accumulate under disease conditions such as cancer [5]. These cells inhibit immune effectors through various mechanisms. Specifically, MDSCs can directly inhibit the growth and proliferation of T cells by inducing the differentiation of Treg cells through secretion of interleukin (IL)-10 and transforming growth factor-β [5,6] or by breaking down L-arginine, which is required for the T cell cycle, using arginase 1 expressed in MDSCs [7]. In addition, T cell activity can be inhibited by nitric oxide (NO) and reactive oxygen species (ROS) produced by inducible nitric oxide synthase (iNOS) and NADPH oxidase 2 (NOX2) expressed in MDSCs [8]. 

MDSCs are a heterogeneous cell population typically classified into two subsets [9,10]. In tumor-bearing mice, CD11b^+^Ly-6G^low^Ly-6C^high^ cells are classified as mononuclear (Mo)-MDSCs and CD11b^+^Ly-6G^high^Ly-6C^low^ cells as polymorphonuclear (PMN)-MDSCs [10]. Recently, other MDSC subsets containing novel functional markers have been suggested [11,12].

Overcoming MDSC-mediated immune suppression is important for successful cancer immunotherapy. This includes the use of various agents that can deplete MDSCs, mainly cytotoxic anti-cancer agents such as gemcitabine [13], cisplatin [14], docetaxel [15], and 5-fluorouracil [16]. In addition, beneficial effects of inhibitors of functional regulators such as NO [8,17], arginase [18], cyclooxygenase2 [19], and ROS [9] have been reported. There are also strategies that have been studied for reducing the immunosuppressive activity of MDSCs by using all-trans retinoic acid (ATRA) [20], IL-12 [21], and CpG [22], which can differentiate MDSCs into macrophages or dendritic cells (DCs). Based on these studies, we investigated the role of antioxidants eliminating ROS (one of the main functional mediators of immune suppression of MDSCs) as a regulator of MDSCs.

ATX is a member of the carotenoid family with a strong antioxidant capacity. It modulates various signaling pathways, such as the extracellular-signal-regulated kinase (ERK), PI3K/Akt, and c-Jun N-terminal kinases pathways [23,24,25,26]. It has been reported to increase the expression of nuclear factor erythroid 2-related factor 2 (Nrf2)-regulated enzymes by inducing nuclear translocation of the transcription factor, Nrf2. This elicits anti-cancer effects by inhibiting cancer cell proliferation and apoptosis induction, by eliminating ROS, and through its anti-inflammatory activity [26]. We predicted that the antioxidant activity of ATX would eliminate the major factors mediating immune suppression of MDSCs, such as ROS, and ATX might alter the viability or function of MDSCs by regulating cell signaling of MDSCs. In addition, ATX is expected to enable further differentiation of MDSCs into macrophages or DCs through the induction of glutathione (GSH) synthesis by activating the Nrf2 signaling pathway in MDSCs. This is because one of the targets of ATX, Nrf2, is a transcription factor of *NQO-1, HO-1, GCLC*, and *GCLM* [27,28]. Among these genes, *GCLC* and *GCLM* might contribute to antioxidant activity as well as cell differentiation through GSH synthesis.

Currently, there is insufficient information on the effect of ATX in immunosuppressive cells such as MDSCs. Through this study, we confirmed that treatment with ATX in vivo and in vitro changed the phenotype of MDSCs, similar to the immune effectors. In addition, the expression of functional mediators and Nrf2 target genes was significantly changed through ATX treatment. ATX not only acts as a direct antioxidant but also induces functional changes in MDSCs. The altered MDSCs are rather immunogenic APCs that activate the T cell response and mediate anti-cancer effects. Overall, the results of this study confirmed the direct and indirect actions of ATX as an antioxidant, as well as its maturation-inducing and function-regulating activity in immune cells. These data suggest the possibility of using ATX as an antioxidant with immunoregulatory functions in cancer therapy.

## 2. Materials and Methods 

### 2.1. Mice and Tumor Model

Specific pathogen free-female BALB/c mice were purchased from Orient bio, Korea. All mice were kept at the Animal Resource Center of Inje University. Experiments were approved by the Institutional Animal Care and Use Committee of Inje University (Approval number: 2017-002). 

Mouse colon tumor cell line, CT26 cells (Korean cell bank) were maintained in Dulbecco’s modified Eagle’s medium (DMEM) supplemented with 10% fetal bovine serum (FBS) and 1% penicillin-streptomycin solution (all from Gibco, Germany). For solid tumor model, BALB/c mice were s.c. injected with 5 × 10⁵ cells/mouse of CT26 at the left flank. Tumor growth was monitored at 2- to 3-day intervals. Tumor size was measured by caliper and was calculated as follows: the longest length × the shortest width × height × π/6.

### 2.2. MDSC Isolation

CT26 tumor-bearing mice were sacrificed at about 40 days after tumor challenges. Splenocytes were prepared, and RBCs were removed using ammonium-chloride-potassium (ACK) lysis buffer (Gibco, USA). Cells were stained with anti-CD11b microbeads (Miltenyi Biotec, Germany), and CD11b^+^ cells were separated using MACS LS column (Miltenyi Biotec, Germany) according to the manufacturers’ recommendation. 

### 2.3. Viability Assay

MDSCs were seeded at 1 × 10⁶ cells/wells in 96-well plate (SPL, Korea) and treated with 100 ng/mL of lipopolysaccharide (LPS, Sigma, USA) and the indicated concentration of ATX (Adipogen, Switzerland) or dimethyl sulfoxide (DMSO, Sigma, USA) as vehicle (veh). After 24 h incubation, 20 μL/well of thiazolyl blue tetrazolium bromide (MTT, Sigma, USA) was added to MDSCs. After 2 h in a humidified atmosphere, insoluble crystals were detected. After centrifugation of the plate, the media were removed and formazan crystals were solubilized in DMSO. Absorbance of samples at 570nm was measured using microplate reader Sunrise™ (Tecan, Austria). 

### 2.4. Phenotype Analysis of MDSCs

CT26 tumor-bearing mice with about 100 mm³ of tumor size were administrated with 50 mg/kg of ATX or veh, olive oil (Sigma, USA) using sonde for 10 days daily. Splenocytes were obtained, and some cells were stained with anti-CD11b microbeads to MDSC isolation. After MACS separation, cells were stained with fluorescein isothiocyanate (FITC)-labeled anti-Ly-6G Abs and phycoerythrin (PE)-labeled anti-Ly6C Abs for MDSC gating. For analysis of MDSC phenotype, we used allophycocyanin-labeled anti-CD40 Abs, anti-CD80 Abs, anti-CD86 Abs, or anti-IA:IE Abs. Other splenocytes were stained with allophycocyanin-labeled anti-F4/80 Abs or anti-CD11c Abs. For T cell analysis, cells were stained with FITC-labeled anti-CD3 Abs and either PE-labeled anti-CD4 Abs or PE-labeled anti-CD8 Abs. For Treg staining, cells were fixed and permeabilized using fix/perm kit (ebioscience, CA) and stained with allophycocyanin-labeled anti-Foxp3 Abs (All from BioLegend, CA).

For in vitro ATX treatment, MDSCs were seeded at 2 × 10⁷ cells/well in 6-well cell culture dish (SPL, Korea) and incubated in the presence of 10 ng/mL of granulocyte-macrophage colony-stimulating factor (GM-CSF, BioLegend, USA) for 5 days. ATX (10 μM) or veh, DMSO was added to MDSCs on day 0 and day 3. After incubation, cells were harvested and stained with fluorescent-labeled Abs. Stained cells were analyzed by flow cytometry (FACSCalibur, BD Science, USA). 

### 2.5. Real Time-quantitative Polymerase Chain Reaction (RT-qPCR) 

Isolated MDSCs were seeded at 10⁷ cells/well in 12-well cell culture plate (SPL, Korea) and treated with ATX or veh in the presence of 100 ng/mL of LPS for 24 h or 5 days. After incubation time, cells were harvested and RNA was purified using RNeasy mini kit (Qiagen, Germany). We used M-MLV cDNA Synthesis Kit for cDNA synthesis and TopReal™ qPCR Kit (Both from Enzynomics, Korea) for RT-qPCR. The following primers (All from Cosmogenetech, Korea) were used: Glyceraldehyde 3-phosphate dehydrogenase (*GAPDH*), Forward 5′–CCT GGA GAA ACC TGC CAA GTA–3′, Reverse 5′–GGA AGA GTG GGA GTT GCT GTT G–3′, Arginase 1 (*ARG1*), Forward 5′–AAC ACG GCA GTG GCT TTA ACC T–3′, Reverse 5′–GTG ATG CCC CAG ATG GTT TTC–3′, NADPH oxidase 2 (*NOX2*), Forward 5′–GAC CCA GAT GCA GGA AAG GAA–3′, Reverse 5′–TCA TGG TGC ACA GCA AAG TGA T–3′, Inducible Nitric Oxide Synthase (*INOS*), Forward 5′–AGG AAG TGG GCC GAA GGA T–3′, Reverse 5′–GAA ACT ATG GAG CAC AGC CAC AT–3′, NAD(P)H: quinone oxidoreductase 1 (*NQO1*), Forward 5′–GCA TTG GCC ACA CTC CAC CAG–3′, Reverse 5′–AGT GCC CAC AGA GAG GCC AAA–3′, Hemox1 (*HO-1*), Forward 5′–CAC GCC AGC CAC ACA GCA CTA–3′, Reverse 5′–GGC TGT CGA TGT TCG GGA AGG–3′, Glutamate-Cysteine Ligase Catalytic Subunit (*GCLC*), Forward 5′–ACA TCT ACC ACG CAG TCA AGG ACC–3′, Reverse 5′–CTC AAG AAC ATC GCC TCC ATT CAG–3′, Glutamate-Cysteine Ligase Modifier Subunit (*GCLM*), Forward 5′–GGC TTC GCC TCC GAT TGA AGA–3′, Reverse 5′–TCA CAC AGC AGG AGG CCA GGT–3′. 

### 2.6. ROS Detection

MDSCs were treated with 10 μM of ATX in the presence of LPS (100 ng/mL). After treatment, MDSCs were harvested via centrifugation and resuspended in 10 μM of CM-H2DCFDA (Invitrogen, USA) for 50 min according to the manufacturers’ recommendation. Cells were analyzed by flow cytometry. 

### 2.7. In Vivo CTL Assay

MDSCs were treated with ATX or veh for 24 h. MDSCs were pulsed with Her-2/neu CTL epitope peptides p63 (Anygen, Korea) [29] at a concentration of 5 μg/mL for an additional 90 min. Cells were harvested and transferred into naïve BALB/c mice via intravenous (i.v.) route. After 2 weeks, we performed in vivo CTL assay as previously described [30]. For preparation of Her-2/neu-specific target cells, naïve splenocytes were pulsed or unpulsed with 5 μg/mL of p63 peptides for 90 min. Peptide pulsed cells were labeled with 20 μM of carboxyfluorescein diacetate succinimidylester (CFSE, Invitrogen, USA), whereas peptide unpulsed cells were labeled with 1.5 μM of CFSE. The same amount of peptide pulsed or unpulsed cells were mixed. Target cells (1 x 10⁷cells/mouse) were i.v. injected into mice. After 72 h, p63-specific target lysis in the splenocytes was analyzed by flow cytometry. The specific lysis was calculated as follows: *r* = (% CFSE^low^ cells/% CFSE^high^ cells), % specific lysis = [1 − (*r*_unprimed_/*r*_primed_)] × 100.

### 2.8. Adoptive Transfer of MDSCs

Isolated MDSCs were seeded at 10⁷ cells/well in 12-well cell culture plate and treated with ATX or veh for 24 h. For maintenance of immunosuppressive MDSC function, MDSCs were incubated in Roswell Park Memorial Institute Medium 1640 (RPMI1640, Gibco BRL, Germany) supplemented with 10 ng/mL of GM-CSF, 20% of FBS, and 25% of tumor cell conditioned medium (TCCM) which were the supernatants of CT26 cell culture media [31]. AXT-treated or veh-treated MDSCs (1 × 10⁷ cells/mouse) were i.v. transferred into CT26 tumor-bearing mice at the tumor size of about 100 mm³. Tumor size was monitored 3 times a week. We monitored tumor size until day 42 after tumor challenge.

### 2.9. Statistical Analysis

The student’s *t*-test was used to compare the differences between 2 groups. Values of *p* < 0.05 were considered significant at a 95% confidence interval.

## 3. Results

### 3.1. ATX Injection Induces Immunogenic Conversion of Mo-MDSCs with a Decreasing Percentage of PMN-MDSCs in Tumor-Bearing Mice

**Figure 1:**
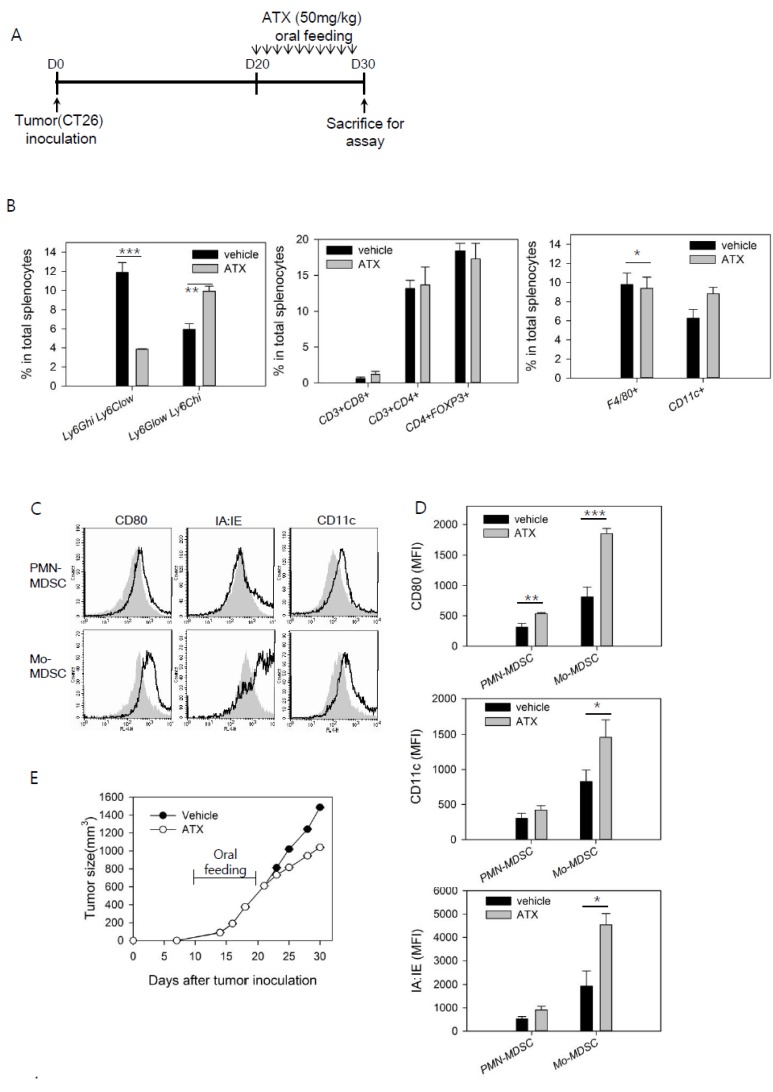
Changes in the percentages and phenotype of myeloid-derived suppressor cells (MDSCs) are induced in tumor-bearing mice treated with astaxanthin (ATX). Tumor-bearing mice were treated orally with ATX (50 mg/kg) daily for 10 days (n = 4/group). After treatment, mice were sacrificed and their splenocytes were stained with fluorescent-labeled Abs. For analysis of MDSCs, CD11b^+^ cells were separated from splenocytes, then stained with fluorescent-labeled Abs. Splenocytes were analyzed by flow cytometry. (**A**) Experimental schedule for ATX treatment of tumor-bearing mice. (**B**) Percentages of Ly-6G^high^Ly-6C^low^ polymorphonuclear (PMN)-MDSCs and Ly-6G^low^Ly-6C^high^ mononuclear (Mo)-MDSCs (left). Percentages of CD8^+^ T cells, CD4^+^ T cells, and FoxP3^+^ regulatory T (Treg) cells (middle). Percentages of F4/80^+^ cells and CD11c^+^ cells (right). (**C**) Surface markers on PMN-MDSCs and Mo-MDSCs. Histograms show data of veh-treated mice as filled areas and ATX-treated mice as bold lines. (**D**) Mean fluorescence intensities (MFIs) of MDSCs are shown as means ± SEM (n = 3). (**E**) Tumor size was monitored at 2-3 day intervals. Representative data from two independent experiments. * *p* < 0.05, ** *p* < 0.01, *** *p* < 0.001.

To determine the antitumor effect of ATX in tumor-bearing mice after feeding them ATX (50 mg/kg) for 10 days (Figure 1A), the growth of a solid tumor and differentiation of immune cells in the mice were analyzed simultaneously (Figure 1B–E). The percentages of myeloid cell and T cell subsets in the spleen were analyzed; no significant changes in CD4^+^ T cells, Treg cells, or CD8^+^ T cells were found. However, the percentage of CD11b^+^Ly-6G^high^Ly-6C^low^ PMN-MDSCs was considerably decreased, while the percentage of CD11b^+^Ly-6G^low^Ly-6C^high^ Mo-MDSCs was significantly increased (Figure 1B). 

Levels of surface markers in the MDSC subsets were determined. The CD80 activation marker in PMN-MDSC was significantly increased. Expression levels of MHC class II molecules (IA:IE) and the DC-specific marker, CD11c, were also increased, in addition to that of CD80 in Mo-MDSCs (Figure 1C,D). The growth of tumor was suppressed in mice fed ATX (Figure 1E). Overall, changes in the number and phenotype of immune suppressor cells, MDSCs, were induced in tumor-bearing mice through ATX injection. Importantly, this treatment showed an antitumor effect.

### 3.2. In Vitro Treatment with ATX Induces MDSC Differentiation

**Figure 2:**
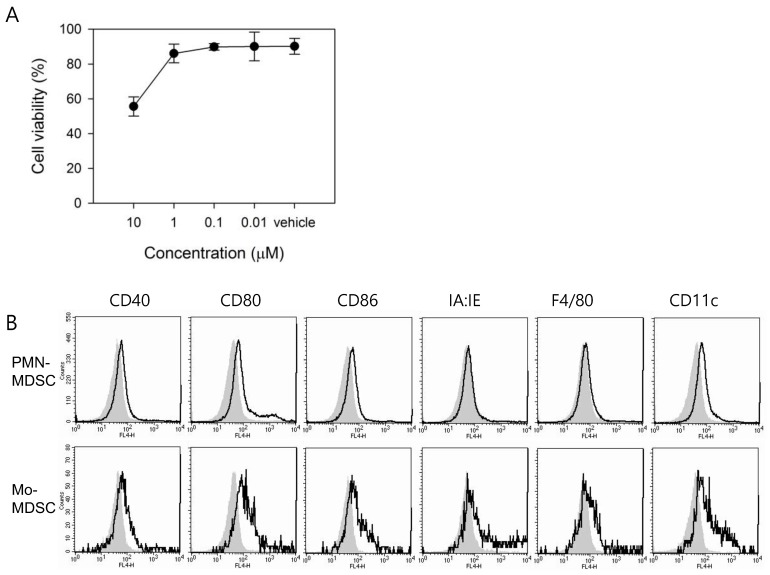
In vitro treatment with ATX induces MDSC differentiation. (**A**) MDSCs were isolated from spleens of CT-26 tumor-bearing mice, and the cells incubated for 24 h in the presence of ATX and LPS. Cell viability was measured by the thiazolyl blue tetrazolium bromide (MTT) assay. Data are shown as means ± SEM. Data are representative of two experiments. (**B**) MDSCs were isolated from tumor-bearing mice and cultured for 5 days with 10 ng/mL of GM-CSF. ATX or veh was added on days 0 and 3. After treatment, cells were harvested and stained with fluorescent-labeled Abs. The levels of surface markers, such as CD40, CD80, CD86, IA:IE, F4/80, and CD11c were measured on each MDSC subset. Data show veh-treated (filled areas) and ATX-treated (bold lines) cells. Data are representative of three experiments.

A significant increase of the DC marker, CD11c, in the spleen after in vivo ATX treatment was observed. Considering the hypothesis that MDSCs with immature characteristics can undergo further differentiation into macrophages or DCs in response to ATX, we analyzed MDSC viability and the expression of the cell surface markers on isolated MDSCs in the presence of ATX. To measure the effect of ATX on MDSC viability, ATX was diluted from an initial concentration of 10 μM to final concentration of 0.01 μM and subsequently used to treat MDSCs for 24 h. To reconstitute ATX, we used DMSO as the vehicle for in vitro cell treatment, while olive oil was used for in vivo study to exclude the possibility of a toxic effect of DMSO in treated mice. To compare in vitro cell culture concentration with in vivo dose, we have to consider the difference in solubility of ATX in each vehicle and also bioavailability of each formulation. For in vitro maintenance of MDSCs, 100 ng/mL LPS was used. The highest concentration of ATX (10 μM) resulted in a survival rate of about 55 %, and lower concentrations resulted in survival rates above 90 % (Figure 2A). At an ATX concentration of 1 μM, which had a negligible effect on the viability of MDSCs, no significant difference in the expression levels of *ARG1*, *iNOS*, and *NOX2*, which are the functional mediators involved in MDSC-mediated immune suppression, were observed (data not shown). Therefore, subsequent experiments were carried out using a 10 μM concentration of ATX, at which more than half of the ATX-treated cells survived.

To analyze the effect of ATX treatment on the activation/differentiation status of MDSCs, these cells were incubated for 5 days in the presence of GM-CSF with or without ATX (Figure 2B). ATX treatment resulted in more noticeable changes in Mo-MDSCs than in PMN-MDSCs. In particular, expression of the DC marker, CD11c, was increased in Mo-MDSCs compared to expression of the macrophage marker, F4/80. The expression of surface markers, such as CD80, was increased by ATX treatment in both MDSC subsets, consistent with the in vivo experimental data. Collectively, these results confirmed that both MDSC subsets achieved a higher maturation status by inducing differentiation through ATX treatment.

### 3.3. ATX Treatment Reduces the Expression of Functional Mediators of MDSCs and Induces the Expression of Genes Involved in GSH Synthesis

**Figure 3:**
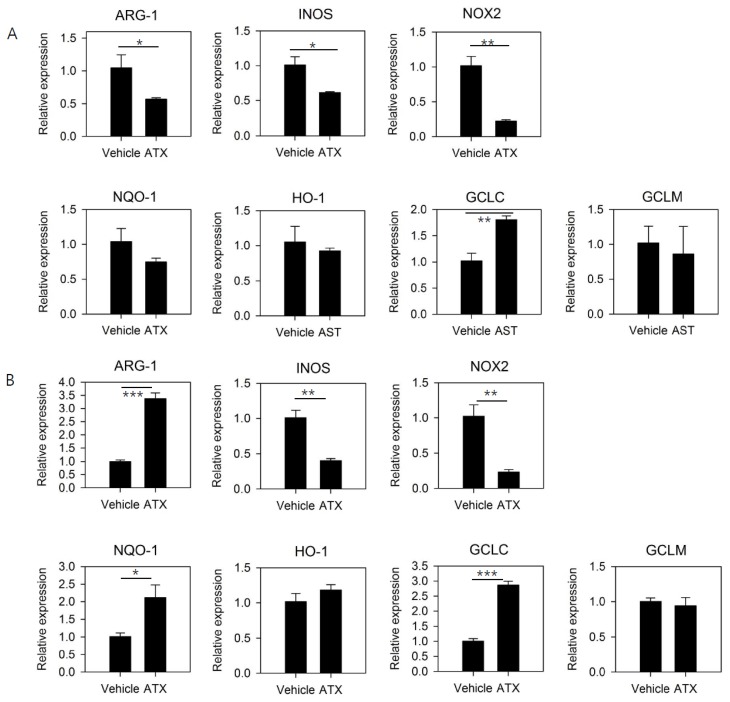
ATX treatment reduces the expression of functional mediators of MDSCs and induces the expression of genes involved in GSH synthesis. MDSCs were isolated from spleens of tumor-bearing mice and cultured for (**A**) 24 h and (**B**) 5 days in the presence of ATX and LPS. RNA was extracted, and expression of the indicated genes was evaluated by the quantitative real-time PCR. Data are presented as means ± SEM of three samples and are representative of at least three experiments. * *p* < 0.05, ** *p* < 0.01, *** *p* < 0.001.

To confirm the mechanism underlying the antitumor effect of ATX, isolated MDSCs were treated with ATX, and changes in gene expression of these cells were analyzed after 24 h (Figure 3A) and 5 days (Figure 3B). The expression levels of *ARG1*, *INOS*, and *NOX2,* which are involved in the immunosuppression mediated by MDSCs, were significantly reduced after 24 h of ATX treatment; however, after 5 days of treatment, significant decreases could be observed in *NOX2* and *INOS* expression but not *ARG1* expression.

ATX regulates various cell signaling pathways, including Nrf2 signaling, which mediates antioxidant activity. ATX treatment resulted in increased induction of *GCLC* with the 24-h ATX treatment and *NQO-1* and *GCLC* with the 5-day ATX treatment. The ATX-mediated inhibition of the immunosuppressive activity of MDSCs may be due to the inhibition of iNOS and NOX2 expression. In addition, the mechanisms underlying ATX-mediated changes in antioxidant activity, and the induction of cell differentiation in MDSCs might correlate with Nrf2 signaling pathway activation. Overall, these gene expression data suggest it is possible to regulate MDSC function by ATX treatment. 

### 3.4. ATX Treatment Reduces ROS Level in MDSCs

**Figure 4:**
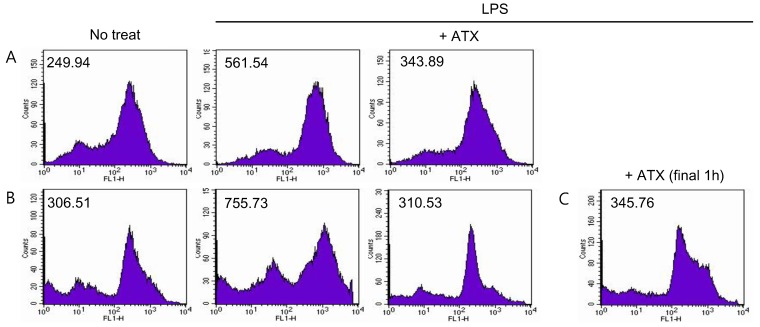
ATX treatment reduces ROS level in MDSCs. MDSCs were isolated from spleens of tumor-bearing mice and cultured for (**A**) 24 h and (**B**) 5 days in the presence of ATX and LPS. (**C**) MDSCs were incubated in LPS containing media for 5 d. At day 5, ATX was added to MDSCs, and then cells were incubated for 1 h. To measure the ROS level in MDSCs, we added CM-H_2_DCFDA to MDSCs and evaluated their fluorescence by flow cytometry. MFIs are shown. Data are representative of two independent experiments.

ATX has a direct antioxidant activity through singlet oxygen quenching or free radical scavenging; this is due to its unique molecular structure. To confirm the direct effect, we measured the changes in ROS levels in ATX-treated MDSCs. To maintain MDSCs and induce ROS generation, 100 ng/mL of LPS was added to the isolated cells and incubated along with ATX for 24 h (Figure 4A) and 5 days (Figure 4B). ROS induced by LPS were significantly decreased by ATX treatment. This decrease can also potentially be induced by an indirect effect of ATX on MDSCs by inducing expression of antioxidant enzymes. To exclude this possibility, we stimulated MDSCs with LPS for 24 h, and for the last hour (after 23 h of LPS stimulation), MDSCs were treated with ATX. ROS levels were measured at the end of the incubation period. The result confirmed the immediate reduction of ROS by ATX (Figure 4C). In conclusion, ATX has antioxidant activity and this action might help regulate the immunosuppressive functions of MDSCs.

### 3.5. ATX-Treated MDSCs Act As Immunogenic APCs With Antitumor Activity

**Figure 5:**
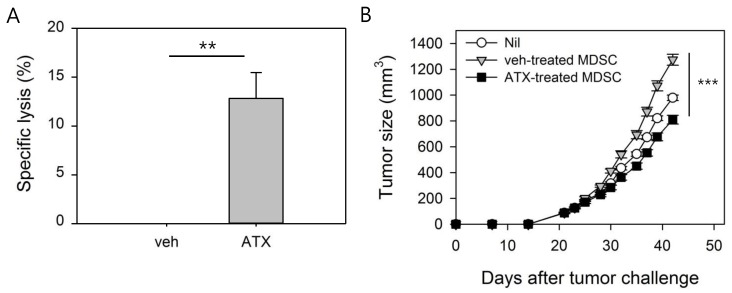
ATX-treated MDSCs act as immunogenic antigen-presenting cells (APCs) with antitumor activity. (**A**) MDSCs were isolated from spleens of tumor-bearing mice and cultured for 24 h in the presence of ATX or veh. Then, Her-2/neu CTL peptide, p63, was pulsed to veh-treated MDSCs and ATX-treated MDSCs for 90 min. Each of these MDSCs were injected intravenously into naïve mice (n = 3/group). After 2 weeks, CFSE-labeled, p63-pulsed syngeneic splenocytes were transferred into MDSCs-injected mice. After 72 h, mice were sacrificed and CFSE^+^ cells in splenocytes were analyzed by flow cytometry. Data are presented as means ± SEM of three mice and are representative of two experiments. **p < 0.01. (**B)** MDSCs were isolated from spleens of tumor-bearing mice and treated with ATX or veh for 24 h in the presence of TCCM and GM-CSF. Then, MDSCs were adoptively transferred via intravenous injection to tumor-bearing mice (n = 5/group). Tumor size was monitored at 2- to 3-day intervals. *** *p* < 0.001.

We next determined whether changes in the phenotype and gene expression in MDSCs in response to ATX treatment mediated the functional changes. To determine the immunogenicity of ATX-treated MDSCs, CTL epitope peptide of Her-2/neu (p63), a well-known tumor-associated Ag, was pulsed into MDSCs that were injected into naïve mice. Analysis of the in vivo CTL response induced by P63-pulsed MDSCs showed that control MDSCs, with poor immunogenic characteristics, did not induce an immune response, while ATX-treated MDSCs induced Ag-specific lysis (Figure 5A).

MDSCs are a key factor in an immunosuppressive tumor environment and have tumor promotion characteristics [32,33]. To determine whether ATX-treated MDSC loses its tumor-promoting activity, adoptive transfer to tumor-bearing mice was performed and tumor growth was monitored (Figure 5B). In this experiment, TCCM was added to maintain the immunosuppressive function of MDSC during the 24 h in vitro incubation process, and GM-CSF was added to maintain the MDSCs. Intravenous injection of MDSCs in mice with tumors approximately 100 mm³ in size revealed that the tumor size in mice given veh-treated MDSCs was increased compared to the nil group, presumably because of the tumor-promoting activity of MDSCs, while the tumor-promoting activity was not observed in mice given ATX-treated MDSCs. In conclusion, the in vivo experiment confirmed that MDSCs are converted into immunogenic APCs, and their immune-suppressing property is inactivated by ATX treatment.

## 4. Discussion

Recently, various strategies targeting MDSCs have been verified in preclinical studies and clinical trials [12,34]. One of the major immunosuppressive mechanisms of MDSCs is mediated by ROS, and therefore studies on the functional regulation of MDSCs using antioxidants have been conducted. The immunosuppressive activity of MDSCs on T cells was reduced when ROS levels were decreased using antioxidants [8]. Furthermore, suppression of cancer metastasis through attenuation of ROS production using a synthetic Nrf2 inducer, 1-(2-cyano-3-,12-dioxooleana-1,9(11)-dien-28-oyl) imidazole (CDDO-Im), in MDSCs has been reported [35]. On the contrary, MDSC expansion can be induced through Nrf2-dependent activation, and Nrf2 pathway contributes to defense mechanisms against oxidative stress exposed to MDSCs [36]. Thus, the Nrf2 pathway can either positively or negatively regulate MDSC-mediated immune suppression.

In this study, ATX, a potent antioxidant with anti-cancer effects against various types of cancer cells, was investigated for MDSC targeting. It was hypothesized that the ROS level decreased in ATX-treated MDSCs because of the antioxidant activity of ATX, thereby regulating immune suppression and simultaneously inducing changes via Nrf2 signaling activation. In particular, we focused on *GCLC* and *GCLM* among the Nrf2 target genes [37]. The synthesized GSH is involved in cell differentiation and antioxidant activity [38]. Thus, we believed that ATX not only played a role as a direct/indirect antioxidant but may also induce maturation of MDSCs. This would be significant because differentiated MDSCs may function as immune effectors with reduced immunosuppressive activity [39].

Previously, ATRA and CpG were used to study the differentiation of MDSCs into macrophages or DCs. ATRA induces the synthesis of GSH synthase through ERK signaling pathway in MDSCs, followed by their differentiation into macrophages or DCs with increased GSH [20]. CpG induces MDSC maturation via the activity of interferon-α produced in plasmacytoid DCs [22]. In this study, a significant decrease in the percentage of PMN-MDSCs, and an improved activation status of the remaining MDSCs, was observed in tumor-bearing mice treated with ATX, confirming a significant increment in CD11c^+^ cells. In addition, it was confirmed that in vitro ATX treatment increased the expression of *GCLC*, a gene involved in GSH synthesis, in isolated MDSCs. As a result, the immunogenicity of ATX-treated MDSCs with altered functions was improved. Further, these cells had the characteristics of mature effector cells that exhibit a cancer growth inhibitory effect.

## 5. Conclusions

The strong antioxidant activity of ATX can weaken immunosuppressive function through reduction of ROS levels in MDSCs. In addition, the ROS in MDSCs play a role in maintaining immature status by inhibiting MDSC maturation [40]. Thus, ATX is an agent that convert MDSCs into mature myeloid cells. 

This study indicates that MDSCs become immunogenic following ATX treatment. It demonstrates the role of ATX as a regulator of the immunosuppressive tumor environment, along with its previously known direct anti-cancer effect. Our results may be helpful for understanding the anti-cancer effects of ATX in an immunocompetent host.
